# *Culicoides* Latreille (Diptera: Ceratopogonidae) taxonomy: Current challenges and future directions

**DOI:** 10.1016/j.meegid.2014.12.018

**Published:** 2015-03

**Authors:** L.E. Harrup, G.A. Bellis, T. Balenghien, C. Garros

**Affiliations:** a Vector-borne Viral Diseases Programme, The Pirbright Institute, Ash Road, Pirbright, Surrey GU24 0NF, UK; b University of Queensland, St Lucia, Brisbane, Qld, Australia; c Cirad, UMR15 CMAEE, 34398 Montpellier, France; d INRA, UMR1309 CMAEE, 34398 Montpellier, France

**Keywords:** *Culicoides*, Biting midge, Arbovirus, Taxonomy, Molecular entomology, DNA barcode

## Abstract

*Culicoides* Latreille biting midges (Diptera: Ceratopogonidae) cause a significant biting nuisance to humans, livestock and equines, and are the biological vectors of a range of internationally important pathogens of both veterinary and medical importance. Despite their economic significance, the delimitation and identification of species and evolutionary relationships between species within this genus remains at best problematic. To date no phylogenetic study has attempted to validate the subgeneric classification of the genus and the monophyly of many of the subgenera remains doubtful. Many informal species groupings are also known to exist but few are adequately described, further complicating accurate identification. Recent contributions to *Culicoides* taxonomy at the species level have revealed a high correlation between morphological and molecular analyses although molecular analyses are revealing the existence of cryptic species. This review considers the methods for studying the systematics of *Culicoides* using both morphological and genetic techniques, with a view to understanding the factors limiting our current understanding of *Culicoides* biology and hence arbovirus epidemiology. In addition, we examine the global status of *Culicoides* identification, highlighting areas that are poorly addressed, including the potential implementation of emerging technologies.

## Highlights

• We review tools for *Culicoides* species identification using both morphological and genetic characterisation.
• We review progress in integrative taxonomy in *Culicoides.*
• We present the current global status of *Culicoides* taxonomic knowledge.
• Present conclusions on the current status in *Culicoides* taxonomy and systematics and prospects for the future.

## 1 Introduction

Biting midges of the genus *Culicoides* Latreille, 1809 (Diptera: Ceratopogonidae) are among the smallest haematophagous flies described, measuring from 1 to 3 mm in body length (Mellor et al., 2000). The genus receives considerable attention through the role of several species as biological vectors of pathogens of medical and veterinary importance. In addition to several nematode and protozoan species, over 50 arboviruses have been isolated from species of *Culicoides* and their role in the transmission of veterinary (Borkent, 2004; Meiswinkel et al., 2004b; Mellor et al., 2000) and human (Carpenter et al., 2013; Linley, 1985) pathogens has been reviewed. Opportunistic feeding of *Culicoides* species on humans can additionally impact upon tourism, forestry and agricultural industries (Mellor et al., 2000). Currently, the greatest economic impact of *Culicoides* lies in their ability to transmit bluetongue virus (BTV), epizootic haemorrhagic disease virus (EHDV) and African horse sickness virus (AHSV). These arboviruses are of significant importance in ruminants, deer and equines, respectively and outbreaks are notifiable to the Office International des Épizooties (OIE) (OIE, 2014). *Culicoides* have also recently been identified as the vector of the novel *Orthobunyavirus*, Schmallenberg virus (Elbers et al., 2013).

In biologically-transmitted, vector-borne pathogens, the phenotypic and genetic traits of the vector(s) play a key role in determining the epidemiology of transmission. Subtle differences in the biology and ecology of closely related species can exert significant effects on the probability of transmission, the most important of which are the ability to become infected with and transmit pathogens (‘vector competence’) and the degree of association with a specific host (‘host preference’). Accurate separation of species is therefore basic to understanding the epidemiology of disease transmission.

The vast majority of taxonomic studies of *Culicoides* rely on morphological analyses although in the nearly thirty years since the classification of the *Culicoides* genus was described as being in disarray (Jones et al., 1985), a substantial decline in the availability of ‘classical’ morphological taxonomic expertise and infrastructure has occurred. The advent of ‘molecular entomology’ for systematics is perceived by many as providing a rapid alternative to the development of classical taxonomic expertise (Tautz et al., 2003) and the genetic characterisation and delimination of species of *Culicoides* via the phylogenetic species concept are increasing in importance. This process has been accelerated by the unprecedented emergence of arboviruses in new regions, requiring rapid characterisation of the local fauna to answer epidemiological questions in the absence of classical taxonomic expertise (Carpenter et al., 2009).

This review considers the taxonomic methods used to identify *Culicoides* from the perspective of both morphological and genetic techniques, with a view to understanding the factors limiting our current understanding of their biology and role in pathogen epidemiology. We additionally examine the global status of *Culicoides* identification, highlighting areas that are poorly addressed including the implementation of emerging technologies that may assist in this process.

## 2 Systematic classification of *Culicoides*

Early checklists of *Culicoides* species, for example Kieffer (1906), were hampered by a lack of clarity regarding generic limits and contained numerous species that have since been removed from the genus and omitted others now regarded as valid members of *Culicoides*. The genera of Ceratopogonidae were reassessed in subsequent papers by Kieffer (1919, 1925) and Goetghebuer (1920) and adopted by Edwards (1926) and this forms the basis of the modern concept of *Culicoides*.

The first comprehensive worldwide checklist for species of *Culicoides* following stabilisation of the genus, was produced by Arnaud (1956) and subsequently updated by Arnaud and Wirth (1964). A later list provided by Boorman and Hagan (1996) did not identify synonyms, so their total number did not reflect valid species but Borkent and Wirth (1997) provided a comprehensive list of valid names which has been maintained online by Borkent (2014b) to date. Linked to the latter publication, Borkent (2014a) provides the only comprehensive list of subgeneric placements of species of *Culicoides* which, despite not strictly following the most recent valid combinations from the literature nor listing where transfers were actioned, is an invaluable resource.

### 2.1 Subgeneric classification

The current subgeneric classification of *Culicoides* consists of 31 subgenera (Table 1) containing 63% of extant species, 38 unplaced groups of species containing 24% of extant species and a further 13% of extant species that workers have not been able to place into any of these groupings (Borkent, 2014a). Species of economic importance are placed into a wide variety of these subgeneric groupings although some, for example *C.* subg. *Avaritia*, appear to contain a larger proportion of vector species than most other groupings (Meiswinkel et al., 2004a; Wirth and Dyce, 1985). The lack of vector competence data for most species of *Culicoides* does not, however, allow conclusions to be drawn on how vector competence for the different *Culicoides*-borne viruses have evolved within the genus.

Contributions to the subgeneric classification of *Culicoides* have in general been based on regional assessments with limited attempts to rationalise groupings with those from other regions (Fox, 1948, 1955; Khalaf, 1954; Root and Hoffman, 1937). There is mounting evidence to suggest that at least some of the current subgenera are polyphyletic i.e. derived from more than one common ancestor (Gomulski et al., 2006; Linton et al., 2002; Perrin et al., 2006; Schwenkenbecher et al., 2009a) while others are probably synonymous. A major consequence of such poorly defined subgenera is the frequent disagreement on the placement of some species. For example, Yu et al. (2005) placed *C. nudipalpis* Delfinado, 1961 into *C.* subg. *Culicoides* based on the absence of a palpal pit while other workers, using a suite of other characters, have consistently placed this species into *C.* subg. *Avaritia* (Bellis et al., 2014a; Dyce et al., 2007; Meiswinkel and Baylis, 1998; Meiswinkel et al., 2004a; Wirth and Hubert, 1989)*.* Similarly, *C.* subg. *Oecacta* is often used as a dumping ground for species authors have trouble placing elsewhere (Boorman, 1988; Wirth and Dyce, 1985).

Borkent (2014a) noted that the subgeneric classification of *Culicoides* is almost entirely phenetic i.e. based on morphological similarity, generally of adult specimens, although a very limited number of studies have also included characteristics of immature stages in their assessments but with variable success (Glukhova 1977; Nevill and Dyce, 1994; Nevill et al., 2009). Cladistic studies, using morphological comparison with outgroups to identify taxa with shared synapomorphies, have been used with some success to validate the status of several genera of Ceratopogonidae (Borkent, 2000), but the subgeneric groupings within *Culicoides* are largely untested (Borkent 2014a) leading to the instability noted above*.*

Few workers have attempted to utilise genetic data and phylogenetic analysis to test subgeneric groupings of *Culicoides* (Bellis et al., 2014a; Pagès et al., 2009). At a higher level, only a single study has used genetic data to test the higher classification of Ceratopogonidae, however, support for many of the resulting clades was low and did not support the monophyly of several recognised taxa, including the genus *Culicoides* (Beckenbach and Borkent, 2003).

While the International Code of Zoological Nomenclature (ICZN) provides guidelines for the use and format of formal nomenclature terms including species name and subgenus (ICZN, 2012), the infrasubgeneric categories ‘species complex’ and ‘species group’ have no formal status under the ICZN yet their use is prevalent within the *Culicoides* literature. Borkent (2014a) lists species groups which are currently unplaced to formal subgenera but not those that are placed within a formal subgenus, for example the 10 groupings of species of *C.* subg. *Avaritia* recognised by Meiswinkel et al. (2004a) and Dyce et al. (2007). While many of the species groups listed by Borkent (2014a) are likely to eventually be raised as valid subgenera, as recently occurred with *C.* subg. *Marksomyia* Bellis and Dyce (2011), the groups currently placed within existing subgenera are not likely to be elevated to a formal taxon and are currently referred to as either species groups or species complexes, often interchangeably.

The adoption for *Culicoides* of the standardised use of the informal vernacular names ‘group’ and ‘complex’ as currently used in the Culicidae literature (Harbach, 1994) would go some way to alleviating this problem. Under Harbach’s (1994) scheme, the term ‘species group’ is used to include a collection of related/similar species which exhibit a notable degree of morphological differentiation and the term ‘species complex’ defines a collection of seemingly isomorphic species, which cannot currently be differentiated morphologically in one or both sexes. In contrast to, and in order to differentiate them from species names, names for species complexes and species groups are printed in roman letters with the first letter capitalised i.e. ‘Obsoletus complex’ rather than ‘*C. obsoletus* complex’. For example, the Imicola group consists of 14 morphologically similar and closely related species (Nevill et al., 2007; Bellis et al., 2014a), two of which, *C. brevitarsis* Kieffer, 1917 and *C. bolitinos* Meiswinkel, 1989 are genetically distinct but morphologically indistinguishable as adults (Bellis et al., 2014a) and as pupae (Nevill et al., 2007) and these two species conceivably form the Brevitarsis complex, named after the first described species in the complex.

Where possible, the phylogenetic validity of any species group or complex should be assessed before being proposed to help clarify the placement of species within groups and avoid later confusion. The majority of cryptic species groups/complexes described to date are based on the inability to separate adult, often female, specimens morphologically. Additional diagnostic characteristics are however, often present in immature stages and these may assist in clarifying the status of species with similar adult morphology (Nevill et al., 2007, 2009; Bellis and Dyce, 2011). Harbach (1994) and earlier proponents of the standardised use of the terms species group and species complex (Peyton, 1990), however, do not discuss how these terms should be applied when for example adult forms are isomorphic but diagnostic characteristics are present in larval and/or pupal forms. Ideally all life stages should be studied to reconstruct phyletic lines and grouping (Belkin, 1962), where this is not possible clear statements on which life stages have been considered when proposing new or revising existing groups should be made.

### 2.2 Species recognition

*Culicoides* currently contains 1343 extant and 44 extinct species (Borkent, 2014b), representing the largest genus of the Ceratopogonidae and comprising 21.5% of Ceratopogonid species. As in other Dipteran vector groups, species classification of *Culicoides* has traditionally been dominated by morphological analyses and remains heavily reliant upon the work of individual specialists. Perhaps the best known contributor was Willis Wirth whom, during a prolific career of over 55 years, authored or co-authored more than 400 publications on Dipteran taxonomy. The studies of Wirth and his contemporaries have resulted in a patchy but relatively standardised reporting of the *Culicoides* fauna across a broad global range and major studies addressing specific regions have been produced (Table 2).

In addition to these major taxonomic works are a huge number of other studies of *Culicoides* often with relatively poor descriptions of single or small numbers of species (Boorman, 1988; Crosskey, 1988) many of which do not provide the information required to assess the accuracy of identification. Additionally, many of these studies do not make reference to original type specimens, either due to poor attention to detail on the part of the worker or the loss of type specimens. The latter is a significant problem with older specimens, for example the majority of species described by Jean-Jacques Kieffer (1857–1925), were destroyed by a fire at the Hungarian Natural History Museum (Boros, 1957). An example of the difficulties this creates is *C. oxystoma* Kieffer, 1910 whose original description does not contain sufficient detail to allow separation of this species from others in the Schultzei group, and whose type specimen has been lost (Wirth and Hubert, 1989). This has resulted in the reporting of at least 19 misidentifications and 6 synonymies (Wirth and Hubert, 1989) with these problems continuing to this day e.g. Morag et al. (2012). In these cases the ICZN recommends the designation of neotypes, preferably from the type locality of the species. The increasing number of cryptic species being revealed by genetic analysis (see Section 3.2) emphasises the importance of obtaining material from the type locality as specimens from other regions may eventually be shown to belong to a different species. The loss of type specimens coupled with inadequate descriptions of several other species has led Borkent (2014a) to suggest these are likely *nomen dubia* (ICZN, 2012).

## 3 Tools for *Culicoides* species identification

Species definition of *Culicoides* has traditionally been based on the morphology of adults. Other approaches including the morphology of immatures and genetic characterisation are, however, becoming increasingly common in taxonomic studies and are beginning to reveal deficiencies in the traditional reliance on adult morphology.

### 3.1 Morphological characterisation

#### 3.1.1 Immature *Culicoides*

The morphology of immature specimens has received less attention than that of adults with only 13% of species known as larvae and 17% as pupae (Borkent, 2014a), probably due to the relative difficulty in collecting immatures compared to adults. Immatures are most commonly identified by rearing field-collected specimens to adult and the relative ease of rearing pupae compared to larvae has probably contributed to the higher number of species known as pupae than as larvae.

There are no examples of a species described solely from immature specimens although Nevill et al. (2009) recently provided a description of the pupae of two African species whose adults are yet to be described and are difficult to separate morphologically. Although keys to immature stages of *Culicoides* have been produced for some regional faunas (e.g. Kettle and Lawson, 1952; Howarth, 1985; Elson-Harris and Murray, 1992) the bulk of identification aids for *Culicoides* are based on adult morphology, partially because this is the only means of recognising the bulk of species but also because this is the life stage most commonly encountered either biting hosts or in monitoring programs using light traps.

#### 3.1.2 Adult *Culicoides*

Adult *Culicoides* are notable for their characteristic wing pigmentation pattern and distribution of wing macrotrichia (Table 1), which in certain species can be used as the principle diagnostic feature. Indeed, identification aids based primarily on wing patterns have been published for Nearctic (Wirth et al., 1985), Neotropical (Wirth et al., 1988), Australasian (Bellis et al., 2014b; Dyce et al., 2007), Austro-Oriental (Bellis et al., 2013b) and western Palaearctic faunas (González de Heredia and Lafuente, 2011; Rawlings, 1996). Atypical variants in wing patterns are commonly observed in several species e.g. *C. newsteadi* Austen, 1921 variants N1, N2 and N3 (Pagès et al., 2009), but whether these represent distinct species has yet to be fully resolved. A broad range of other morphological characters of adult specimens are also commonly used including the body and wing size, the distribution, type and number of antennal sensilla, the shape, size and sensilla arrangement of the palpal segments, the number and form of the hind tibial comb, presence of interfacetal hairs, leg colour patterns, the shape and form of the male genitalia and the shape, size and number of the female spermatheca (Cornet, 1974; Wirth and Hubert, 1989).

While the terminology of morphological characters was stable for many years, the relatively recent inclusion in descriptions of multiple forms of antennal sensilla (Cornet, 1974) led to inconsistencies in the terminology of these structures, later standardised by Wirth and Navai (1978). More recently, changes to the naming of some characters, particularly the gonocoxite and gonostylus of the male genitalia and the numbering of antennal flagellar segments, have been adopted by *Culicoides* taxonomists to conform with terminology used in other Diptera (McAlpine, 1981; Teskey, 1981; Merz and Haenni, 2000; Sinclair, 2000).

The largely Wirth-inspired systematic standardisation of characters used to describe *Culicoides* species allowed direct comparison between species and led to the production of identification aids and dichotomous keys to species. These have been successfully applied to a range of regional faunas (Table 2) although little attempt has so far been made to develop keys applicable across classical biogeographic boundaries. More recently, the first open-access interactive key to a regional fauna was developed (Mathieu et al., 2012) heralding a new era in *Culicoides* identification aids. These keys offer a significant improvement on traditional dichotomous keys as they typically provide a suite of illustrations of the important characters for each species and allow the user to select the characters used to progress through the key. Their main disadvantage is the requirement to have a computer alongside the microscope. The lack of agreement on the morphological characters important in defining subgeneric groupings has so far impeded the production of identification aids to these groupings and few keys to regional subgenera have been developed, exceptions include Vargas (1960, 1973a) and Yu et al. (2005).

Traditional morphometrics (Augot et al., 2010; Delécolle, 1985; Pagès et al., 2009) and more recent landmark-based geometric morphometrics (Henni et al., 2014; Muñoz-Muñoz et al., 2011, 2014) have been shown to be useful in enabling the separation of previously intractable specimens. The move from qualitative to quantitative assessments of morphological characters in the latter technique has only been applied to a handful of species but has the potential, in combination with phylogenetic analysis, to provide new insights into *Culicoides* systematics. In practice, however, the requirement for specimens to be slide mounted, image-captured, measured and analysed precludes the use of morphometrics for identification purposes in high-throughput systems such as surveillance programs, even though some aspects of image analysis can be automated (Weeks et al., 1999).

### 3.2 Genetic characterisation

#### 3.2.1 Application of genetic studies to *Culicoides* systematics

The use of genetic data in studies of *Culicoides* systematics is increasing but the scientific rigor with which these studies have been conducted and the phylogenetic analysis interpreted is variable (Garros et al., 2010). Only a small proportion of studies have used multiple molecular markers for species delineation and assessed concordance between the resulting gene trees as performed by Bellis et al. (2014a). Similarly, few studies have compared specimens collected from a sufficiently wide geographic area to confirm that species identifications were conspecific between countries/regions, an exception being the examination of *C. imicola* Kieffer, 1913 by Dallas et al. (2003). In addition, studies have seldom attempted to correlate phylogenetic clades with detailed morphological analysis (Bellis et al., 2013b).

Despite the challenges discussed, the morphological identifications of *Culicoides* by taxonomists have commonly been shown to be concordant with later studies utilising genetic characterisation (Gomulski et al., 2005, 2006; Linton et al., 2002). There are, however, exceptions and the status of some species regarded as conspecific by morphologists may require reassessment given the widely divergent genetics of different populations. An example of this is the presence of cryptic species within the subgenus *Culicoides* in Europe (Gomulski et al., 2006; Pagès et al., 2009). Conversely, some species regarded by morphologists as valid have been unable to be distinguished by genetic analysis e.g. *C. circumscriptus* Kieffer, 1918, *C. manchuriensis* Tokunaga, 1941 and *C. salinarius* Kieffer, 1914 (Ander et al., 2013; Bellis et al., 2013a), while, the specific status of *C. deltus* Edwards, 1939 and *C. lupicaris* Downes and Kettle (1952) is still controversial and needs to be resolved (Meiswinkel et al., 2004a; Nielsen and Kristensen, 2011; Ramilo et al., 2013).

The presence of ‘isomorphic’ species i.e. genetically distinct, morphologically indistinguishable ‘sibling’ species which frequently occur sympatrically, also precludes identification on the basis of morphological characteristics and requires the use of molecular methods for separation. With regards to vector-borne diseases, failure to recognise cryptic species may confound epidemiological investigations due to potential variation in vector competence and/or host preference between sibling species and may complicate control efforts due to variation in the bionomics of the sibling species, for example as observed in the Anopheles Gambiae complex in relation to malaria transmission and control (Collins et al., 2000).

#### 3.2.2 Molecular identification assays in *Culicoides*

To a greater extent than in morphological studies, genetic analyses of *Culicoides* have centred upon putative or confirmed arbovirus vector species. Initial cytotaxonomy studies of *Culicoides* (Hagan and Hartberg, 1986; Isaev, 1982, 1983) were followed by the use of isozyme-based assays in preliminary studies in Europe (Waller et al., 1987) and then addressed cryptic species of the Variipennis complex in the USA (Nunamaker and McKinnon, 1989; Tabachnick, 1992). The latter studies contributed to an eventual systematic reassessment and the raising of several sub-species to full species rank (Holbrook et al., 2000). While isozyme techniques are now less commonly used, they were recently employed in Sardinia (Pili et al., 2010) to investigate population genetic structure in *C. obsoletus* (Meigen) 1818 and *C. scoticus* Downes and Kettle (1952).

Alternative identification methodologies have recently been proposed, including Matrix-Assisted Laser Desorption/Ionization-Time of Flight (MALDI–TOF) analysis (Kaufmann et al., 2012a,b; Kaufmann et al., 2011; Steinmann et al., 2013), however, the benefits of these techniques in reduced cost or labour in comparison to PCR-based assays are yet to be demonstrated. In particular, unlike PCR-base methods, identification profiles for the different life-stages of *Culicoides* are not transferable between life-stages using MALDI–TOF (Kaufmann et al., 2011). While the MALDI–TOF method may have a place within *Culicoides* identification systems, the results produced do not infer any evolutionary information and as such cannot easily be used to further resolve the phylogenetic relationship of the genus.

By far the most commonly employed molecular techniques used to identify *Culicoides* species are those based on polymerase chain reaction (PCR) amplification of DNA. Following pioneering studies (Raich et al., 1993; Sebastiani et al., 2001), sequence analysis of a wide range of mitochondrial DNA (mtDNA) markers has become commonplace and are now the focus of the majority of *Culicoides* phylogenetic studies (Table 3). Recently the fused Carbamoyl phosphate synthetase, Aspartate transcarbamylase and Dihydroorotase (CAD) nuclear marker has also been assessed for its utility in distinguishing species (Table 3). In addition, a microarray hybridization based method based on ITS1 sequence polymorphism has also been utilised for the identification of Palaearctic *C.* subgen. *Avaritia* species (Deblauwe et al., 2012; Augot et al., 2013a) have also developed a Restriction Length Fragment Polymorphism (RLFP) assay for the identification of the morphologically similar species *C. stigma* (Meigen), 1818 and *C. parroti* (Kieffer), 1922.

Direct sequence analysis of molecular markers for *Culicoides* species identification is becoming more common; however logistical and financial constraints still limit their application, particularly in a surveillance role that may require the processing of thousands of individuals. While the use of medium-throughput multiplex PCR assays for the identification of *Culicoides* species has become routine in small to medium sized research projects investigating *Culicoides* abundance, distribution and bionomics in Europe e.g. Harrup et al. (2013) and Viennet et al. (2013), these techniques remain logistically and financially beyond the reach of small, non-specialist research groups. A solution to this bottle neck may lie in the use of quantitative real-time PCR assays for identification of pooled specimens. Four assays of this type have been developed for *Culicoides*. Three of these are based on sequence polymorphism of ITS, the first targeting *C. imicola* (Cêtre-Sossah et al., 2008), the second targeting differentiation of *C. obsoletus* and *C. scoticus* (Mathieu et al., 2011), and a third targeting *C. obsoletus*, *C. scoticus* and *C. montanus* Shakirzjanova, 1962 (Monaco et al., 2010). The fourth assay was based on COI polymorphism and was able to distinguish at least 11 *Culicoides* species or variants (Wenk et al., 2012). While commonly used for quantification and typing of pathogen burdens in both hosts (Mens et al., 2006) and vectors (Hatta et al., 2013). Mathieu et al. (2011) is the first to attempt the use of real-time quantitative PCR techniques for quantification of the relative proportion of species contained within pooled samples for insect species identification purposes and once optimised, this technique should make analysis of large *Culicoides* collections to species-level viable.

While the relative merits of the DNA extraction techniques for a variety of sample types has been previously examined for many types of insect e.g. Wang and Eang (2012), only one study has investigated the impact of DNA extraction technique selection on the sensitivity and specificity of multiplex PCR assay based identification success in *Culicoides* (Garros et al., 2014). Overall, commercial kits where found to produce more consistent results across DNA samples than in-house DNA extraction methods, but the ability for the techniques to be scaled up to at least medium through-put was variable (Garros et al., 2014).

#### 3.2.3 COI and DNA barcoding

There have been several high-profile cases made for a universal DNA-based taxonomic identification system for eukaryotic organisms (Blaxter, 2004; Godfray, 2007; Hebert et al., 2003; Tautz et al., 2002, 2003). In response, the use of sequence diversity in the 5’ section of the COI gene region (defined relative to the mouse mitochondrial genome as the 648 bp region that starts at position 58 and stops at positive 705) has been selected as the focus of a ‘DNA barcoding’ system for animal life (Hebert et al., 2003).

A major advantage of the Consortium for the Barcode of Life (CBOL) initiative (Ratnasingham and Hebert, 2007) lies in the requirement to maintain a set of metadata standards (Hanner, 2012; Ratnasingham and Hebert, 2007). The requirement to upload trace files for sequence data records in particular allows external validation of sequence data quality. This is a prerequisite for records submitted to the Barcode of Life Database (BOLD) to achieve Barcode status (Ratnasingham and Hebert, 2007), but not yet a requirement of GenBank (Federhen et al., 2009). The increasing emphasis on producing at least partially validated sequence data is a step towards reference sequences for species and to a degree combats the proliferation of unauthenticated sequence data held on GenBank which, where investigated, has been found to be unreliable (James Harris, 2003; Nilsson et al., 2006). Conflict between species assignments and genetic similarities have already been identified for several *Culicoides* entries in GenBank (Ander et al., 2013; Bellis et al., 2013a), which could be the result of either misidentifications, evidence of synonymous species or an inability of COI analyses to separate these species. The CBOL initiative also emphasises the importance of retaining morphological ‘vouchers’ for all sequenced specimens as these constitute an essential link between the genetic data and the taxonomic classification enabling verification of identifications and cross-referencing of any subsequent specimens (Pleijel et al., 2008).

Several attempts have been made to clarify relationships and develop identification tools for species of *Culicoides* based on the COI marker, for which both universal (Folmer et al., 1994) and genus-specific (Bellis et al., 2013b; Dallas et al., 2003) primers have been designed and utilised in population and genetic studies e.g. Nolan et al. (2004). Many of these studies, however, have utilised primers which produce truncated sequences shorter than the 500 bp required to achieve Barcode status (Hanner, 2012; Ratnasingham and Hebert, 2007). While there is good evidence to suggest that the COI barcode region is suitable for the discrimination of species of *C.* subg. *Avaritia* (Ander et al., 2013; Bellis et al., 2014a; Jan Debila, 2010; Linton et al., 2002), *C.* subg. *Culicoides* (Ander et al., 2013; Lassen et al., 2012), the Immaculatus group (Bellis et al., 2013a), the Schultzei group (Bakoum et al., 2013; Morag et al., 2012) and the Coronalis, Molestus and Kusaiensis groups (Bellis et al., 2013a), there are indications that variation in this region is insufficient for delimitations of species within at least some other subgenera e.g. *C. *subg. *Beltranmyia* (Ander et al., 2013; Bellis, 2013).

#### 3.2.4 ITS

Ribosomal DNA markers have been used to investigate phylogenies (ITS1 and ITS2: Gomulski et al., 2005, 2006; Perrin et al., 2006; 28S: Henni et al., 2014), interspecific genetic distances (ITS1: Nielsen and Kristensen, 2011) and population structure (ITS1: Ritchie et al., 2004) within *Culicoides*. Sequence diversity in the ITS regions has also been used to develop both conventional (Mathieu et al., 2007; Stephan et al., 2009) and real-time (Cêtre-Sossah et al., 2008; Mathieu et al., 2011; Monaco et al., 2010) PCR assays for the identification of BTV vector species from individual specimens and pooled samples.

#### 3.2.5 CAD

An alternative marker which is gaining popularity within insect phylogenetics is the nuclear CAD gene, which is present within Diptera as a single copy (Adams et al., 2000; Freund and Jarry, 1987; Holt et al., 2002). Previous studies in other insect genera have suggested that parts of this gene contain a high number of parsimony informative sites (Moulton and Wiegmann, 2004; Sharanowski et al., 2011; Swaffield and Purugganan, 1997). Portions of the CAD region have increasingly been used to aid resolving of both shallow and deep evolutionary relationships of insect genera e.g. Wiegmann et al. (2009) and Moulton and Wiegmann (2004), and has shown promise as a valuable secondary marker for *Culicoides* studies utilising the COI Barcode region marker (Bellis et al., 2014a; Bellis et al., 2013b).

#### 3.2.6 Other molecular markers

In addition to COI, CAD, ITS1 and ITS2, several additional markers have been utilised on a small-scale in *Culicoides* studies (Table 3). In comparison to other vector groups there has, however, been little investigation into other marker regions. Several markers which have previously been utilised for Dipteran phylogenetics but which have not yet been utilised for *Culicoides* studies include the small ribosomal subunit (12S), elongation factor-1α (EF-1α), NADH dehydrogenase subunit 2 (ND2), NADH dehydrogenase subunit 4 (ND4), NADH dehydrogenase subunit 5 (ND5), NADH dehydrogenase subunit 6 (ND6), 6-phosphogluconate dehydrogenase (PGD), triose phosphate isomerase (TPI) and the *white*, *wingless, cacophony* and *period* genes (Barr and McPheron, 2006; Dyer et al., 2008; Gibson et al., 2011; Lins et al., 2002). Further assessment into relative performance for these markers for the systematics of *Culicoides*, as performed for other insect genera (Baker et al., 2001; Gibson et al., 2010; Wild and Maddison, 2008), and an assessment of the ease of amplification and sequencing of these regions is required before firm recommendations on their utility for *Culicoides* systematics can be made.

## 4 Phylogenetics to phylogenomics

With increasing technical capabilities to produce sequence data and corresponding increases in computational capacity, phylogenetic analysis is beginning to enter a new era, moving from the analysis of evolutionary relationships on the basis of single genes or small numbers of genes towards the use of genome-scale (Stencel and Crespi, 2013) datasets to infer evolutionary relationships (Barrett et al., 2013; Brito and Edwards, 2009; Narum et al., 2013; Xi et al., 2012), so called ‘phylogenomics’ (Delsuc et al., 2005). Following on from initial preliminary physical mapping of the *Culicoides* genome (Nunamaker et al., 1999) progress began in 2013 on producing the first annotated whole genome dataset for a species of *Culicoides* (Fife et al., 2013; Nayduch et al., 2014), targeting the American BTV vector *C. sonorensis* Wirth and Jones, 1957. In addition to aiding the design of appropriate primers for other gene regions not yet investigated in *Culicoides*, the publication of this first *Culicoides* genome represents a vital step to increasing the ease in which additional *Culicoides* species genomes can be assembled, annotated and incorporated into future phylogenomic studies. The usefulness of these datasets for taxonomic purposes was recently demonstrated with the production and analysis of reference genome assemblies for 16 species of *Anopheles* (Neafsey et al., 2013) which have revealed discord between the relative placement of Anopheles complex species based on phylogenomic data (Besansky, 2014) in comparison to relationships based on more limited numbers of genetic markers (Obbard et al., 2009).

## 5 Combining morphological and genetic resources

The use of molecular data has rejuvenated interest and activity in systematics (Pires and Marinoni, 2010). Although the use of morphological data remains prevalent within insect systematics (Bybee et al., 2010), the common misconception that molecular data can replace the role of classical taxonomy has been repeatedly refuted in the literature (Carvalho et al., 2007; Ebach and Holdrege, 2005; Will et al., 2005; Will and Rubinoff, 2004). It is therefore essential that ‘classical’ morphological taxonomy be integrated with high-quality phylogenetic analysis, at least in initial instances.

Dayrat (2005) sets out guidelines for the level of support required prior to the creation of new species including the incorporation of genetic information in the taxa description. The existence of isomorphic species, however, questions the requirement for a morphological description for every new species. Cook et al. (2010) argued that new species descriptions need not be accompanied by morphological confirmation and proposed a protocol for describing new species based solely on genetic data. While this has not yet occurred for any species of *Culicoides*, Bellis et al. (2014a) recently confirmed the existence of two isomorphic species in this genus, *C. brevitarsis* and *C. bolitinos*, indicating that the protocols offered by Cook et al. (2010) may eventually be required.

Obtaining genetic data from holotype specimens would seem a logical progression in defining a species and should be encouraged whenever new species are described. While the retrieval of viable genetic material retrospectively from slide-mounted *Culicoides* type specimens may prove impossible, in these cases, the next best approach is to utilise specimens from the type locality of the species (Bellis et al., 2013b).

The recent increase in availability of relatively low-cost digital imaging systems for stereo- and compound microscopes, in combination with software for the analysis of images, has greatly enhanced the acquisition of high-quality images of key diagnostic characteristics from voucher specimens. The cost of good quality imaging systems, however, is still prohibitive for some laboratories and despite the increase in the ease with which high-quality digital images of diagnostic characteristics can be acquired, the benefits of traditional high-quality drawings to illustrate morphological variation should also not be over looked, for example see Bellis and Dyce (2011). The widespread availability of internet connectivity in addition to digital imaging facilities also enables an unprecedented level of connectivity between ecologists, taxonomists and any other workers with an interest in *Culicoides*, enabling second opinions to be readily sought and material compared without the need to physically move specimens between labs (Ang et al., 2013; Beaman and Cellinese, 2012; Smith and Blagoderov, 2012), which is particularly important where movement of specimens is problematic due to biodiversity legislation (Schindel, 2010). Bioinformatics tools for the free online dissemination of linked morphological and genetic data exist via BOLD (Ratnasingham and Hebert, 2007), and data from several *Culicoides* studies have already been deposited and made publicly available via BOLD (Bellis et al., 2013a, 2013b, 2014a). GenBank also provides the utility, although more limited than that of BOLD, to link morphological specimen data to sequence data records (Federhen et al., 2009).

### 5.1 Progress in integrative taxonomy in *Culicoides*

Datasets of sequences for national checklists of *Culicoides* morphospecies are starting to be published (Ander et al., 2013; Matsumoto et al., 2009b; Slama et al., 2014), although often with a limited number of specimens per morphospecies. A dramatic increase in the number of specimens analysed per morphospecies and the range of geographic areas from which these samples are collected is required in order to investigate within-species haplotype diversity and reassess species deliminations using cladistic analysis.

There is clearly a need for combining morphological and genetic analyses in taxonomic studies of *Culicoides* with the assessment of congruence between morphological and molecular phylogenetic data, so called ‘integrative taxonomy’ (Will et al., 2005), providing independent confirmation of species definitions. Studies integrating detailed morphological examinations with phylogenetic analysis have already yielded important information regarding the subgeneric classification of *Culicoides* and the identification of new species (Bellis et al., 2014a; Bellis et al., 2013b).

The association of sex and life history stages is a prerequisite to understanding the bionomics of a species, but as a result of the difficulties in sampling and defining diagnostic morphological characteristics of the juvenile life-stages of *Culicoides,* integrative taxonomic efforts have nearly entirely been driven by examination of adult specimens e.g. Bellis et al. (2014a) and Pagès and Sarto i Monteys (2005). To date, only a handful of studies have utilised molecular techniques to aid identification of immature *Culicoides* specimens (Schwenkenbecher et al., 2009b; Swanson, 2011; Yanase et al., 2013). It is this area, however, that molecular tools perhaps have the most to offer with regard to identifying conspecific life-stages and sexes of specimens for which morphological identification is either difficult or impossible (Bellis et al., 2013b). The use of molecular data has also proven useful for the identification of *Culicoides* specimens which have been damaged during collection preventing conclusive morphological identification (Bellis et al., 2013a).

### 5.2 Specimen selection for integrative taxonomic studies

Selection of specimens in integrative taxonomic studies is almost always based initially on morphological examination. The development of comprehensive identification aids based on morphology should therefore be the first step in embarking on these studies. After compiling a checklist of species likely to be present, a key designed to assist with sorting unmounted specimens such as those produced by Goffredo and Meiswinkel (2004), Rawlings (1996) and Bellis et al. (2014b) will help workers to assign tentative names to the specimens they encounter. While taxonomic literature can still be challenging to obtain, the increasing availability of electronic resources now enables rapid collating of documents (Table 4). Alternative sampling strategies for the collection of specimens may also be required in addition to collections made via light trapping e.g. Capela et al. (2003), Goffredo and Meiswinkel (2004) and Venail et al. (2012). Of paramount importance is to collect specimens from as wide a geographic and ecological range as possible to maximise the chances of sampling the full range of genetic variability within a species. Simply increasing sampling size will not necessarily increase the percentage of total haplotypes discovered (Zhang et al., 2010), as haplotype diversity may vary across a species’ geographic range and between ecosystems utilised by the species (Frézal and Leblois, 2008; Sperling, 2003). This was recently demonstrated when Bellis et al. (2014a) investigated the genetic differences between populations of *C. brevitarsis* at the extremities of this species’ distribution which proved to belong to different species.

### 5.3 Vouchering of selected specimens

Definitive identification of species using morphology requires that specimens be slide-mounted in permanent media (Boorman, 1988; Borkent et al., 2009). Although previously popular, pin mounting of *Culicoides* is not suitable for specimen preservation due to their fragile nature (Foote and Pratte, 1954). Standardised methods for slide mounting dissected adult *Culicoides* are available (Boorman, 1988; Boorman and Rowland, 1988; Clastrier, 1984; Wirth and Marston, 1968) and preparation of immature specimens for slide mounting are discussed by Kettle and Lawson (1952). C*ulicoides* specimens mounted whole are not suitable for detailed study of morphological characteristics and should be avoided wherever possible (Boorman, 1988). While gum/chloral media may provide rapid mounts, methods such as that described by Campbell and Pelham-Clinton (1960) should not be used for *Culicoides* voucher specimens as these mounts can darken with age (Boorman, 1986; Boorman, 1988). Care must be taken not to introduce artefactural variation into specimens as a result of the slide mounting process (Arnqvist and Martensson, 1998; Dujardin et al., 2010).

The recent development of a non-destructive DNA extraction technique for use with *Culicoides* (Bellis et al., 2013b) has the advantage of producing entire, cleared specimens ready for slide mounting and has made it possible to develop a library of sequence data with matched high-resolution digital images of slide-mounted morphological voucher specimens. Importantly, it also allows for the deposition of mounted holotype specimens alongside corresponding DNA data (Bellis et al., 2014a). Previous studies have required the use of destructive (Augot et al., 2010) or semi-destructive (Stephan et al., 2009; Slama et al., 2014) DNA extraction protocols with only partial specimens then available for morphological examination. Where semi-destructive protocols are followed there is a need to preserve the important morphological features of voucher specimens; most of the important body parts needed for identification are on the head, abdomen, wings and legs and these should, where possible, be retained. Standardised methodology for the collection of matched morphological and COI Barcode data are now also available online (Harrup, 2014).

## 6 The current global status of *Culicoides* taxonomic knowledge

The biological sciences are facing a tremendous loss of classical morphological taxonomic expertise (Wilson, 1985), the so called ‘taxonomic impediment’ (Tautz et al., 2003). Developments in molecular species delimination and identification methods are, however, progressing at such a pace that there is a danger that the link to expertise in field-based bionomics investigations and morphological taxonomy will be lost. Contributing to this is a lack of support for traditional morphological studies by funding bodies who tend to favour research using new technologies. It is as a response to this that an ‘integrative’ future for *Culicoides* taxonomy should be promoted, where molecular, ecological and morphological investigations are closely linked e.g. Holbrook et al. (2000) and Meiswinkel and Linton (2003).

The major challenges facing *Culicoides* taxonomists include the small size of specimens, a poorly defined subgeneric classification, the lack of descriptions for conspecific life-stages and sexes, the lack of phylogenetic data, intra-specific variation in diagnostic morphological characters, the identification and resolution of potentially synonymous species and a lack of consensus on defining appropriate intraspecific genetic distances. Without a resolution to these problems we are unable to accurately delineate the geographical range of many species and have limited information on the regional occurrence and temporal abundance patterns of the majority of species. Currently the effort expended on *Culicoides* taxonomy globally is uneven with areas serviced by taxonomists or which suffer veterinary/public health implications exhibiting high species richness while other areas where high levels of biodiversity would be expected, have relatively few *Culicoides* species recorded, for example in the Neotropics. This is highly likely to be as a result of sampling bias rather than a true measure of biodiversity. For example the *Culicoides* fauna of Madagascar is probably largely underestimated given its size and habitat diversity, having been subject to only limited study (Augot et al., 2013b; De Meillon, 1961; Kremer et al., 1972).

The ability to accurately identify specimens from large-scale surveillance projects would enable investigations into relationships between species richness and climate, latitude, landcover, topography, host availability and seasonality (Andrew et al., 2013). International collaboration for knowledge exchange and technology transfer, in addition to the utilisation of online open-access systems for both morphological and molecular taxonomic information, is essential if the regional variations in species delimitations and identification are to advance and coalesce (Ang et al., 2013). This is particularly the case in response to the proliferation of species which has occurred as a result of recent research in the oriental region (Yu et al., 2005).

Specific taxonomic questions which still require addressing include the species *C. oxystoma* and members of the Schultzei group (Bakoum et al., 2013; Boorman, 1988), *C. lupicaris* and *C. deltus* (Downes and Kettle, 1952; Glukhova and Leningrad, 1989; Gomulski et al., 2006; Kettle and Lawson, 1952), and whether species with wide geographic ranges are in fact conspecific. Examples of the latter include *C. montanus* described both in Europe and Central Asia (Gomulski et al., 2006; Shakirzjanova, 1962), *C. obsoletus* recorded in the Palaearctic, Nearctic and Oriental regions (Arnaud, 1956; Boorman, 1988), *C. circumscriptus* in the Palaearctic and Oriental regions (Arnaud, 1956; Kremer and Delécolle, 1974) and *C. punctatus* (Meigen), 1804 which exhibits a >12% sequence divergence between European and Japanese specimens (Matsumoto et al., 2009a). Whether differences such as those evident in *C. punctatus* represent the existence of multiple species or a gradation in genetic diversity across a geographical gradient remains to be seen. In contrast, specimens of *C. imicola* from the two extremities of its distribution i.e. the Republic of South Africa and eastern China, have an identical COI haplotype (Bellis et al., 2014a).

## 7 Conclusions and prospects for the future

DNA characterisation provides a new means and impetus to understand morphological and functional disparity within the genus *Culicoides*. Previous studies have demonstrated congruence between morphological and molecular analysis (Gomulski et al., 2005; Pagès et al., 2009; Pagès and Sarto i Monteys, 2005) indicating that when used in tandem these techniques can help resolve species complexes and investigate phenotypic plasticity. Their use in attempts to unravel morphologically similar species has begun, for example within the subgenus *Avaritia* (Bellis et al., 2014a; Mathieu et al., 2007; Pagès and Sarto i Monteys, 2005).

This review has summarised the concepts and techniques that underlie *Culicoides* taxonomy and concludes with the following wish-list for methodologies and resources for integrative taxonomy within the genus: (i) the proliferation of validated multi-marker phylogenetic information with sound phylogenetic analysis of the resulting data; (ii) the continued maintenance of a catalogue of *Culicoides* species, their synonyms and subgeneric placement, which accurately reflects the latest valid treatment in the literature and provides detail on the history of subgeneric placements; (iii) real-time multiplex PCR assays for isomorphic species identifications; (iv) cost-effective next generation sequence analysis pipelines suitable for use with *Culicoides* data; (v) standardisation of morphological terminology and characters used for species and subgeneric descriptions; (vi) user-friendly, well-illustrated taxonomic keys with an evolution away from the traditional dichotomous keys towards interactive Bayesian keys; (vii) landmark-based geometric morphometrics; (viii) testing of the current subgeneric classification system using molecular and/or morphological cladistic analyses; and (ix) the integration of novel taxonomic techniques into large-scale surveillance schemes. Support and recognition by funding bodies and institutions of the importance of having a stable and well resolved taxonomy for *Culicoides* and other significant pathogen vectors is also utmost if research into vector-borne diseases is to progress beyond the use of model species (Agnarsson and Kuntner, 2007; Besansky et al., 2003; Godfray, 2007; Mallet and Willmott, 2003; Wilson, 2003).

A cladistic reinterpretation of the subgeneric classification of *Culicoides* and species delimitations based on molecular phylogenetic analyses with phenotypic and bionomic data overlaying the cladograms, should be the ultimate goal of *Culicoides* taxonomy. While this may seem a distant target, the increasing use of molecular techniques to investigate the evolutionary history of *Culicoides* will allow some aspects of *Culicoides* species delimination and relationships to be resolved. Such resolution will allow for the investigation of co-evolution of vector-host and vector-pathogen systems to begin (Jaafar et al., 2014), an aspect of *Culicoides* biology that has to date received little attention and for which a stable and well resolved classification of the *Culicoides* genus is indispensable.

## Figures and Tables

**Table 1:** Subgenera of *Culicoides* as recognised by Borkent (2014a) with wing pattern of representative species morphological characteristics.

Subgenus	Type species	Number of extant species	Wing pattern of representative species†
*Amossovia* Glukhova *in*Glukhova and Leningrad (1989)	*C. dendrophilus* Amosova, 1957	12	*C. dendrophilus* Amosova, 1957 ♀
*Anilomyia*Vargas (1960)	*C. covagarciai* Ortiz, 1950	19	*C. decor* (Williston), 1896 ♀
*Avaritia*Fox (1955)	*C. obsoletus* (Meigen), 1818	103	*C. obsoletus* (Meigen), 1818 ♀
*Beltranmyia*Vargas (1953)	*C. crepuscularis* Malloch, 1915	43	*C. crepuscularis* Malloch, 1915 ♀
*Cotocripus*Brèthes (1912)	*C. caridei* (Brèthes), 1912 (=*Cotocripus caridei* Brèthes)	6	*C. caridei* (Brèthes), 1912 ♀
*Culicoides* Latreille in Mirzaeva and Isaev (1990)	*C. punctatus* (Meigen), 1804	56	*C. punctatus* (Meigen), 1804 ♀
*Diphaomyia*Vargas (1960)	*C. baueri* Hoffman, 1925	22	*C. baueri* Hoffman, 1925 ♀
*Drymodesmyia*Vargas (1960)	*C. copiosus* Root and Hoffman, 1937	25	*C. loughnani* Edwards, 1922 ♀
*Fastus* Liu in Yu and Huang (2006)	*C. alpigenus* Yu and Liu, 2006	11	*C. erairai* Kono and Takahasi, 1940 ♀
*Glaphiromyia*Vargas (1960)	*C. scopus* Root and Hoffman, 1937	3	*C. scopus* Root and Hoffman, 1937
*Haemophoructus*Macfie (1925)	*C. maculipennis* (Macfie), 1925 (= *Haemophoructus maculipennis* Macfie)	16	*C. maculipennis* (Macfie), 1925 ♀
*Haematomyidium*Goeldi (1905)	*C. paraensis* (Goeldi), 1905 (= *Haematomyidium paraensis* Goeldi)	39	*C. debilipalpis* Lutz, 1913 ♀
*Hoffmania*Fox (1948)	*C. insignis* Lutz,1913 (*=C. inamollae* Fox and Hoffman 1944)	82	*C. peregrinus* Kieffer*,* 1910 ♀
*Jilinocoides*Chu (1983)	*C. dunhuaensis* Chu, 1983	4	♦
*Macfiella*Fox (1955)	*C. phlebotomus* (Williston), 1896 (*=Ceratopogon phlebotomus* Williston*)*	2	*C. phlebotomus* (Williston), 1896 ♀
*Marksomyia*Bellis and Dyce (2011))	*C. marksi* Lee and Reye, 1953	6	*C. marksi* Lee and Reye, 1953 ♀
*Mataemyia*Vargas (1960)	*C. mojingaensis* Wirth and Blanton, 1953	19	*C. mojingaensis* Wirth and Blanton, 1953
*Meijerehelea*Wirth and Hubert (1961)	*C. guttifer* (de Meijere), 1907 (= *Ceratopogon guttifer* de Meijere)	11	*C. guttifer* (de Meijere), 1907 ♀
*Monoculicoides*Khalaf (1954)	*C. nubeculosus* (Meigen), 1830 (= *Ceratopogon nubeculosus* Meigen)	23	*C. nubeculosus* (Meigen), 1830 ♀
*Nullicella*Lee (1982)	*C. lasaensis* Lee, 1978	1	♦
*Oecacta*Poey (1853)	*C. furens* (Poey), 1853 (= *Oecacta furens* Poey)	178	*C. furens* (Poey), 1853 ♀
*Pontoculicoides* Remm *in*Remm and Zhogolev (1968)	*C. tauricus* Gutsevich, 1959	14	Δ
*Psychophaena*Philippi (1865)	*C. venezuelensis* Ortiz and Mirsa, 1950 (= *Psychophaena pictipennis* Philippi)	2	*C. venezuelensis* Ortiz and Mirsa, 1950 ♂
*Remmia*Glukhova (1977)	*C. schultzei* (Enderlein), 1908 (= *Ceratopogon schultzei* Enderlein)	8	*C. oxystoma* Kieffer, 1910 ♀
*Selfia*Khalaf (1954)	*C. hieroglyphicus,* Malloch, 1915	7	*C. hieroglyphicus* Malloch, 1915 ♀
*Silvaticulicoides*Glukhova (1977)	*C. fascipennis* (Staeger) 1839 (= *Ceratopogon fascipennis* Staeger)	12	*C. fascipennis* Kieffer, 1919 ♀
*Sinocoides*Chu (1983)	*C. hamiensis* Chu, Qian and Ma, 1982	8	♦
*Synhelea*Kieffer (1925)	*C. tropicalis* Kieffer, 1913	34	*C. tropicalis* Kieffer, 1913 ♀
*Trithecoides*Wirth and Hubert (1959)	*C. flaviscutatus* Wirth and Hubert, 1959	60	*C. fulvithorax* (Austen), 1912 ♀
*Tokunagahelea*Dyce and Meiswinkel (1995)	*C. mikros* Dyce and Meiswinkel, 1995	3	*C. mikros* Dyce and Meiswinkel, 1995 ♀
*Wirthomyia*Vargas (1973b)	*C. segnis* Campbell and Pelham-Clinton, 1960	8	*C. segnis* Campbell and Pelham-Clinton, 1960 ♀

^∗^An additional 324 extant species are currently placed within 38 species groups that are unplaced within the current subgeneric classification of *Culicoides*, with an additional 173 extant species simply placed within a miscellaneous group and 5 unplaced extant species listed as *nomina dubia* (Borkent, 2014a).

† Wing images not shown to scale.

Δ No wing photograph available see Mathieu et al. (2012) for photograph of wing patterns characteristic of this subgenus.

♦ No wing photograph available see Yu et al. (2005) for drawings of wing patterns characteristic of this subgenus.

**Table 2:** Identification aids to the *Culicoides* fauna by biogeographical region as defined by Holt et al. (2013).

Biogeographical region	Keys to the *Culicoides* fauna
Afrotropical and Madagascan	Boorman and Dipeolu (1979, Nigeria), Cornet and Brunhes (1994, Schulzei group, Africa), Glick (1990, Kenya), Khamala and Kettle (1971, eastern Africa), Meiswinkel (1995, Imicola group of *C*. subg. *Avaritia*), Meiswinkel and Dyce (1989, *C*. subg. *Synhelea*, Africa), Nevill and Dyce (1994, Simils group pupae, Africa), Nevill et al. (2007, Imicola group of *C*. subg. *Avaritia* pupae, Africa)

Australasian and Oceania	Bellis et al. (2013b, Immaculatus group, Australasia; 2014a, Imicola group of *C*. subg. *Avaritia*, Australasia; 2014b, Northern Territory, Western Australia and South Australia), Bellis and Dyce (2011, *C*. subg. *Marksomyia*, Australasia; 2012, Bancrofti group, Australasia), Dyce et al. (2007, Australasia and Oceania), Dyce and Meiswinkel (1995, *C*. subg. *Tokunagahelea*, New Guinea), Dyce and Wirth (1997, Purus group, Australasia, Indonesia), Kettle and Elson (1976, Australian pupae), Lee and Reye (1953, Australia), Tokunaga (1959, 1962a, 1963, 1976, New Guinea), Tokunaga and Murachi (1959, Micronesia), Wirth and Arnaud (1969, Polynesia), Elson-Harris and Murray (1992, Australian pupae)

Nearctic	Atchley (1967) (New Mexico, USA; 1970, *C*. subg. *Selfia*, Nearctic), Atchley and Wirth (1979) (Haematopotus group, Nearctic), Blanton and Wirth (1979, Florida, USA), Downes and Wirth (1981, Nearctic), Foote and Pratte (1954, eastern USA), Fox (1955, Nearctic), Holbrook et al. (2000, Variipennis complex of *C*. subg. *Monoculicoides*, Nearctic), Jamnback (1965, New York, USA), Jones and Wirth (1978, Stonei group, Nearctic), Macfie (1948, Chiapas, Mexico), Root and Hoffman (1937, North America), Wirth (1952, California, USA), Wirth et al. (1985, Nearctic), Wirth and Blanton (1967, Guttipennis group, Nearctic; New World; 1969, Pulicaris group, Nearctic; 1974, Caribbean), Wirth and Hubert (1962, Piliferus group, New World), Wirth and Rowley (1971, Palmerae group, New World)

Neotropical and Panamanian	Aitken et al. (1975, Trinidad and Tobago), Brickle and Hagan (1999, Belize), Felippe-Bauer et al. (2003, Paraensis group, Amazonian region; 2013, *C*. subg. *Mataemyia*, Neotropical), Matta (1967, El Salvador and Honduras), Rodriguez and Wirth (1986, Columbia), Santarém et al. (2014, Reticulatus group, Neotropical), Spinelli et al. (1993, Guttatus group of *C*. subg. *Hoffmania*, Neotropical; 2005, Argentina), Spinelli and Martinez (1991, Uruguay), Williams (1956, Bermuda), Wirth and Blanton (1959, Panama; 1968, Hylas group; 1974, Caribbean), Wirth et al. (1988, Neotropical)

Oriental and Sino-Japanese	Arnaud (1956, Japan and Korea), Bellis (2014, Indian *C*. subg. *Avaritia*), Delfinado (1961, Philippines), Howarth (1985, Lao PDR adults and pupae), Hubert and Wirth (1961, Okinawa, Japan), Kitaoka (1977, Nansei Islands, Japan; 1985a,b, Japan), Lien et al. (1997, 1998, Taiwan), Sen and Das Gupta (1959, India), Takahashi (1941, 1958, Japan), Tokunaga (1940, Japan, China, Micronesia, Mongolia; 1962b, Ryukyu Islands, Japan), Wada (1986, Claggi group, Japan; 1990, Verbosus group, Japan; 1999 Japan), Wirth and Hubert (1959, *C*. subg. *Trithecoides*, Oriental and Afrotropical; 1961, Taiwan; 1989, Southeast Asia), Yu et al. (2005, China)

Palaearctic and Saharo-Arabian	Boorman (1989, Arabian Peninsula), Boorman and van Harten (2003, Yeman), Campbell and Pelham-Clinton (1960, United Kingdom), Delécolle (1985, Northeastern France), Dzhafarov (1964, Transcaucasus), Edwards et al. (1926, 1939, United Kingdom), Glukhova (2005, Russia and adjacents lands), Glukhova and Braverman (1999, Langeroni group), Glukhova and Leningrad (1989, USSR), Goffredo and Meiswinkel (2004, Italy), González de Heredia and Lafuente (2011, Spain), Gutsevich (1966, Central Asia), Kettle and Lawson (1952, United Kingdon pupae), Kremer (1965, France), Mathieu et al. (2012, Western Palaearctic), Rawlings (1996, Iberian Peninsula, Europe), Shahin (1977, Southwestern Asia)

**Table 3:** Molecular markers used for phylogenetic analysis within *Culicoides*.

Genomic region	Molecular marker	References
Mitochondrial	COI	Ander et al. (2013), Augot et al. (2010, 2013a,b), Bakoum et al. (2013), Bellis (2013), Bellis et al. (2013a,b, 2014a), Cêtre-Sossah et al. (2008), Dallas et al. (2003), Dik et al. (2014), Henni et al. (2014), Jan Debila (2010), Lassen et al. (2012), Lehmann et al. (2012), Linton et al. (2002), Matsumoto et al. (2009a), Meiswinkel and Linton (2003), Morag et al. (2012), Muñoz-Muñoz et al. (2014), Nolan et al. (2007), Pagès et al. (2009), Ramilo et al. (2013), Schwenkenbecher et al. (2009a), Swanson (2011), Wenk et al. (2012), Yanase et al. (2013)
	COII	Beckenbach and Borkent (2003), Hey et al. (2003), Matsumoto et al. (2009a)
	28S	Henni et al. (2014)
	18S rRNA	Kiehl et al. (2009)
	16s rRNA	Jan Debila (2010), Meiswinkel and Linton (2003)
	Cyt*b*	Augot et al. (2013a)

Ribosomal	ITS1	Augot et al. (2013a), Cêtre-Sossah et al. (2004), Li et al. (2003), Mathieu et al. (2007), Matsumoto et al. (2009b), Morag et al. (2012), Nielsen and Kristensen (2011), Perrin et al. (2006), Ritchie et al. (2004), Schwenkenbecher et al. (2009a), Stephan et al. (2009), Yanase et al. (2013)
	ITS2	Gomulski et al. (2005, 2006), Matsumoto et al. (2009b), Monaco et al. (2010), Schwenkenbecher et al. (2009a)

Nuclear	CAD	Bellis (2013), Bellis et al. (2013b, 2014a)

**Table 4:** A compilation of online taxonomic tools and resources available for use by the *Culicoides* worker.

Name	Description	Available from
Armed Forces Pest Management Board Literature Retrieval System	An extensive online collection of scientific papers relevant to pathogen vectors including many older and out-of-print journal articles, reports and book sections	http://www.afpmb.org/content/welcome-literature-retrieval-system/
AVAbase (Arthropod Vector of Interest for Animal Health Database)	A multilingual online database of information of arthropod vectors of medical and veterinary importance maintained by Cirad	http://avabase.cirad.fr/
*Culicoides.net*	A resource maintained by The Pirbright Institute, aims to collate and update information on *Culicoides* species recorded in northern and western Europe and other areas with ongoing collaborations	http://www.culicoides.net
DrawWing	A free software package for the description and illustration of insect wings, the associate website also hosts a gallery of wing images for various genera	http://www.drawwing.org/
FLYTREE	Official project webpage of the NSF Assembling the Tree of Life Project EF-0334948 Building the Dipteran Tree of Life: Cooperative Research in Phylogenetic & Bioinformatics of True Flies (Insecta: Diptera), checklists of Ceratopogonid taxa and *Culicoides* subgeneric affiliations	http://wwx.inhs.illinois.edu/research/flytree/
IIKC	An interactive identification key for female *Culicoides* (Diptera: Ceratopogonidae) from the West Palaearctic region (Mathieu et al., 2012)	http://www.iikculicoides.net/
India Bluetongue Vector Network	Official project website of the India Bluetongue Vector Network (IBVNet), hosts standardised protocols for the production of matched morphological voucher specimens and COI Barcode data for *Culicoides*	http://www.ibvnet.com
The Ceratopogonidae Information Exchange (CIE)	A bi-annual newsletter and website hosted by Belmont University providing a directory of Ceratopogonid Workers, brief articles and links to recently published literature of relevance to the Ceratopogonidae fauna	http://campus.belmont.edu/cienews/cie.html/
The Dipterists Forum	A website and forum affiliated to the British Entomological and Natural History Society (BENHS) hosting a photo gallery and copies/links to literature of relevance to the Diptera fauna.	http://www.dipteristsforum.org.uk/
VectorBase	Bioinformatics resource for invertebrate vectors of human pathogens	https://www.vectorbase.org/
